# The Increase of Intra-Abdominal Pressure Can Affect Intraocular Pressure

**DOI:** 10.1155/2015/986895

**Published:** 2015-01-14

**Authors:** Ilhan Ece, Celalettin Vatansev, Tevfik Kucukkartallar, Ahmet Tekin, Adil Kartal, Mehmet Okka

**Affiliations:** ^1^Department of Surgery, Selcuk University, Faculty of Medicine, 42075 Konya, Turkey; ^2^Department of Surgery, Konya Medicana Hospital, 42100 Konya, Turkey; ^3^Department of Surgery, Necmettin Erbakan University, Faculty of Medicine, 42080 Konya, Turkey; ^4^Department of Eye Surgery, Necmettin Erbakan University, Faculty of Medicine, 42080 Konya, Turkey

## Abstract

*Objective*. This study aims to explore the usage of intraocular pressure measurements as the early indicator of the increase in intra-abdominal pressure. *Methods*. In this prospective study, 40 patients undergoing elective surgery were included. Patients were divided into four groups of 10 patients. The control group (Group C) was not subjected to laparoscopic intervention. Laparoscopic surgery was, respectively, performed with an intra-abdominal pressure of 9, 12, and 15 mmHg in Groups L (low), M (medium), and H (high pressure). Intraocular pressure was measured binocularly in each patient at three different times (before, during, and end of surgery) using a contact tonometer. *Results*. Patients' gender, age, body mass index (BMI), American Society of Anesthesiology (ASA) class, and operative times were not different among the groups. No complications occurred with either the surgery or measurement of intraocular pressure. Intubation was associated with a severe rise in IOP (*P* < 0.05). An increase in intraocular pressure was seen in groups M and H (*P* < 0.05). *Conclusion*. Intraocular pressure was increased in the groups with an intra-abdominal pressure of 12 mmHg or more. Measuring the intraocular pressure might be a useful method to estimate the intra-abdominal pressure. This trial is registered with NCT02319213.

## 1. Introduction

The adverse effects of intra-abdominal hypertension (IAH) on other systems were first revealed with the recognition of the relationship between IAH and oliguria. The influence of IAH on cardiovascular, renal, and the pulmonary system became definite through the publications in the first half of the 20th century [1].

The normal pressure of the abdominal cavity is almost equal to atmospheric pressure. However, the pressure increases 5–7 mmHg with the respiratory cycle. Pressures which constantly develop above 12 mmHg are defined as IAH.

Intra-abdominal pressure might be measured invasively through a port placed in the abdomen, the transvesical route, the transgastric route, or the inferior vena cava. The transvesical method is the most common one as it costs less, and it might be easily applied and learned [2, 3]. Measurement with transgastric and vena cava ports has not been commonly used due to the high costs and the infection risk. It has been reported that intrathoracic pressure, intracerebral pressure [4, 5], and intraocular pressure [6, 7] increase when IAP increases. Nevertheless, it is known that increased intracranial pressure would affect intraocular pressure [8]. However, there have not been adequate studies about the relationship between IAP and IOP.

In our study, we aimed to determine the IAP increase by means of measuring the IOP as an alternative to transvesical measurement and to introduce a more practical measurement method for clinical use. The advantages of IOP measurement are the absence of infection risk, easy comprehension, and the nonexistence of costs other than tonometry.

## 2. Methods

After the approval of the institutional Ethics Committee (no. 08-38) and obtaining of informed consent, 40 adult patients between 18 and 55 years old with a body mass index (BMI) of 30 kg/m^2^ or less were included in the study in accordance with the American Society of Anesthesiologists (ASA) I-II status. Exclusion criteria were preexisting eye disease, cardiovascular or neuromuscular disease, difficult intubation, and the use of any antihypertensive agents. A complete ophthalmologic evaluation of each patient was performed before the surgery by the same physician from the Ophthalmology Department.

The patients were divided into four groups with 10 subjects in each. The control group consisted of the patients who had undergone a surgery for inguinal hernia. The study groups were comprised of patients who had undergone laparoscopic cholecystectomy, which allows direct and the most accurate intra-abdominal pressure monitorization. In these cases, the pressure values were monitored from the insufflator monitor and the abdomen was insufflated with 9 mmHg (*n* = 10), 12 mmHg (*n* = 10), and 15 mmHg (*n* = 10). IAP in control group patients was accepted as “zero” in the measurements made without insufflation. Measurement of IOP was performed for both eyes three times: firstly (time 1): postanesthesia induction and 30 seconds prior to the intubation, secondly (time 2): 1 minute after the intubation, and lastly (time 3): 45 minutes after the insufflation. IOP was measured by the same physician from the Ophthalmology Department using the Perkins Hand-Held Applanation Tonometer (Clement Clarke International Limited, England).

### 2.1. Surgical Technique

In control group, patients underwent inguinal hernia repair. In study groups, intra-abdominal insufflation was performed with an infraumbilical 1 cm incision, and all laparoscopic cholecystectomies were performed by a single experienced surgeon with four ports.

### 2.2. Anesthesia Protocol

A standard anesthesia protocol and mechanical ventilation settings were applied to all patients in order to eliminate the anesthesia-related changes. An anaesthesia monitor (Infinity Vista XL, Drägerwerk AG & Co, Germany) was used for standard monitoring including electrocardiography, pulse oximetry, heart rate, noninvasive blood pressure, and neuromuscular transmission.

Patients who had received premedication the previous night and one hour before the operation were administered 1 mcg/kg Remifentanil, 0.5 mg/kg Atracurium, 1-2 mg/kg Propofol for induction, and 0.25 mcg/kg Remifentanil. Additionally, 1 MAC desflurane was used for maintenance of anesthesia. Standardization was attempted by keeping the respiratory rate at 10–12/min, the tidal volume at 8–20 mL/kg, PEEP at 3 cmH_2_O, and EtCO_2_ at 35–40 mmHg on mechanical ventilation (Dräger Primus, Drägerwerk AG & Co, Germany).

### 2.3. Statistical Analysis

The data were evaluated by using the SPSS v13.0 program (SSPS, Inc., Chicago, IL, US). A normality analysis was performed. As the data were consistent with a normal distribution, variance analysis was used for the repeated measurements and the post hoc Tukey test was used for multiple comparisons. The Bonferroni correction *t*-test was used in the dependent groups in order to determine the difference between the measurements. A *P* level of <0.05 was accepted as it was statistically significant.

## 3. Results

The mean age of 40 patients (26 males, 14 females) was 41.4 ± 8.2 years (range 19–55). The mean operative time was 54.2 ± 11.6 minutes and the ASA scores were I and II. The body mass index was 25.3 ± 1.8 (22–30) kg/m^2^. There was not any statistical difference between the groups in terms of age, sex, and BMI. The demographic data are shown in Table 1.

The control group consisted of patients who had undergone an operation for inguinal hernia. All the patients in the study group underwent standard four-port laparoscopic cholecystectomy. There were no complications from the surgery or measurement of IOP. As the IOP values of the right and left eyes did not show a statistically significant difference, analysis was performed on 40 measured values.

The mean IOP in the four groups showed similar values before intubation (time 1) and a similar rise after endotracheal intubation (time 2) (6.4 ± 3.7 mmHg). Furthermore, 12 mmHg or more pressured (Groups M and H) pneumoperitoneum induction led to a significant rise in IOP averaging 8.5 ± 3.4 mmHg. The IOP values of medium and high pressure group at all the measurement points (times 2-3) were higher than the preintubation levels (time 1). However, the IOP values of groups C and L and time 3 measurements were, respectively, decreased from 27% to 25% compared with the time 1 measurements (Table 2). Time 1, IOP values were not different between the male and female patients. In group C and group L, a mild IOP increase was observed at preintubation (time 1) and postinsufflation (time 3) measurements.

## 4. Discussion

Prevalence of IAH has been reported as 18–58.8% regardless of the cause of hospitalization in patients hospitalized in the intensive care unit [7]. IAH must be detected in the early stage in order to eliminate the end organ damage in all internal and surgical clinical disciplines [9, 10]. Many operative and nonoperative methods have been proposed to decrease the abdominal pressure in patients with high intra-abdominal pressure [11–16]. Directly, intra-abdominal measurement of IAP provides the most accurate results. However, it is not practical method for a patient hospitalized in the intensive care unit as it is an invasive method, and it carries the risk of infection. In our study, the desired IAP value was obtained by adjusting the pressure from the insufflator monitor. Therefore, the results of the study were rendered more reliable as the IAP was directly monitorized.

Indirect measurement of IAP might be performed through several methods [17]. The most commonly used measurement method is the transvesical method described by Kron et al. [18]. Several manometers enabling constant measurement and monitorization have been developed; however, these methods have been insufficient to find an adequate area of usage. Urinary infection development due to the fluid given into the bladder would negatively affect the prognosis of intensive care patients. We can predict the increase in intra-abdominal pressure of eligible patients admitted to the intensive care unit with constant intraocular pressure measurements; whereby, the transvesical measurement frequency and infection risk can be reduced. Contact tonometers have minimal risk of cross infection but this could be eliminated with the use of newly manufactured disposable probes [19].

In previous studies, IAP was found to be significantly affected by the body mass index and previous operations [20]. In this study, the body mass indexes being close to each other and application of the same surgical procedure are of importance for standardization. In clinical practice, comparison of baseline measurement of intraocular pressure received during hospitalization and subsequent measurements can provide information about the increase of intra-abdominal pressure. In animal and human tests, the intracranial pressure has been observed to increase when the IAP increases [6]. Halverson et al. predicted that 40 minutes were needed for increased IAPs reflecting onto intracranial pressure [7]. Therefore, in our study, the time 3 measurements were performed at 45 minutes.

Elevated intracranial pressure could impair venous blood flow of the eye, and intraocular pressure increases [7, 21]. Lashutka et al. measured the IOP using tonometry in the patients with elevated intracranial pressure and determined 20 cmH_2_O (1 mmHg = 1.36 cmH_2_O) as the limit value. In that study, the intracranial pressures of the patients with 20 cmH_2_O intraocular pressures were found to be above 20 cmH_2_O. The intracranial pressures of the patients whose IOPs were below 20 cmH_2_O were found to be normal [6].

It is known that pathologic changes gradually develop in all of the organs after the IAP exceeds 12 mmHg [17]. In our study, gradual elevation of the time 1 and time 3 IOP values in the groups in which IAP was 12 and 15 mmHg indicates that the thorax, cranium, and the eye are affected. Thus, the basal IOPs of patients admitted to the intensive care unit must be recorded and IAH must be examined if IOP increases during the follow-ups. Since the study was conducted with healthy volunteers, IAP was not elevated above 15 mmHg. For this reason, studies conducted with intensive care unit patients developing ACS and whose IAPs are severely elevated (>20 mmHg) are required to find a cutoff value.

Measurement of the intraocular pressure may be an alternative method to estimate the IAP in order to reduce the frequency of other indirect measurement methods. Although the findings of our study confirm the hypothesis that 12 mmHg and more intra-abdominal pressure values reflect the IOP, further research is needed. The sample size was limited and IAP values were not high, thus limiting the power of the study, because the study was designed on a healthy population in terms of abdominal hypertension. Nevertheless, our preliminary findings should act as a stimulus for further studies involving larger samples and higher intra-abdominal pressure.

## Figures and Tables

**Table 1:** Demographic data of the patients.

	Group C (*n* = 10)	Group L (*n* = 10)	Group M (*n* = 10)	Group H (*n* = 10)	*P*
Age (y)	40.5 ± 12.4	41.0 ± 10.5	43.1 ± 9.6	42.1 ± 10.2	NS
Gender (M/F)	7/3	6/4	7/3	6/4	NS
BMI (kg/m^2^)	25.8 ± 2.1	25.1 ± 1.9	25.5 ± 2.0	24.8 ± 1.6	NS
ASA score (I/II)	4/6	5/5	6/4	5/5	NS
Operative time (min)	56.4 ± 12.4	58.5 ± 10.2	55.5 ± 11.3	52.3 ± 14.3	NS

NS: not significant; BMI: body mass index; ASA: American Society of Anesthesiologists.

**Table 2:** Changes in intraocular pressure of the groups.

	IAP (mmHg)	Time 1	Time 2	Time 3	*P*
Group C (*n* = 10)	0^*^	10.2 ± 1.4	16.2 ± 4.8	11.7 ± 1.8	NS
Group L (*n* = 10)	9	10.9 ± 1.6	16.8 ± 3.6	12.6 ± 2.2	NS
Group M (*n* = 10)	12	9.9 ± 1.5	17.1 ± 4.1	18.2 ± 3.1	0.018
Group H (*n* = 10)	15	10.3 ± 1.4	16.9 ± 3.8	19.1 ± 3.4	0.021

NS: not significant; IAP: intra-abdominal pressure. ^*^Accepted value.

Data are presented as mean ± SD; time 1: 30 seconds before the intubation, time 2: 1 minute after the intubation, and time 3: 45 minutes after the insufflation.
